# Enhanced participation or just another activity? The social shaping of iPad use for youths with intellectual disabilities

**DOI:** 10.1177/1744629520911311

**Published:** 2020-03-25

**Authors:** Charlotta Isaksson, Elisabet Björquist

**Affiliations:** University West, Sweden; University West, Sweden

**Keywords:** domestication, iPad use, participation, staff members, youths with intellectual disabilities

## Abstract

The use of smartphones and tablet devices in activities is believed to have great potential for enhancing the participation of people with intellectual disabilities. However, these technologies, in themselves, do not contribute to participation. What matters is how they are used. Employing the concept of domestication and insights gained from interviews with staff, this article examines conditions for the enhanced participation of youths with intellectual disability and how tablet devices are being integrated into social care settings, in particular.

The findings reveal two approaches to tablet integration. In one approach, tablet use is an organized practice focused on technology acquisition, skills improvement and entertainment. In the other, it is integrated into existing practices as an aid to interpersonal communication. The organized digital activities create conditions for the youths to participate *like* non-disabled peers. The greatest potential for enabling participation *with* each other is when the youths themselves initiate the use of tablets.

## Introduction

The use of tablet devices and smartphones for entertainment, education and especially communication is a central part of the everyday lives of most children and young people (Hemmingsson, 2015). However, access, as well as the ability to use technologies and participate in digital activities, are not equal for all young people. Those with an intellectual disability are among the disadvantaged (Ramsten et al., 2017). In a society where information and communication technologies (ICTs) are increasingly providing channels for activities, this leads to exclusion (Chadwick et al., 2013; Helsper and Reisdorf, 2016; Macdonald and Clayton, 2013; Watling, 2011).

Furthermore, in everyday life, people with intellectual disability lack the opportunity to use these technologies as an aid for enhancing participation and increasing independence (Burckley et al., 2015; McNaughton and Light, 2013). Previous research has shown that the use of tablets and smartphones with internet access and/or custom-designed applications (apps) can provide support for everyday activities and social interaction, as well as communication in different situations (Barlott et al., 2019; Buchholz et al., 2018; Harris, 2010; Näslund and Gardelli, 2013; Ramsten et al., 2018; Stephenson and Limbrick, 2015). For example, an American intervention study showed that the use of a tablet and the Book Creator^1^ app resulted in an 18-year-old young woman with intellectual disability being less reliant on support staff when shopping in grocery stores (Burckley et al., 2015). An Australian study demonstrated that the use of mobile technology made it easier for participants to seek social support from family and friends when on their own. In turn, this gave them more confidence to participate in everyday activities (Darcy et al., 2016). Finally, a Swedish study described how the use of a mobile phone enabled young adults with intellectual disability to play games and listen to music whenever they wanted during the day (Alfredsson Ågren et al., 2018).

Nonetheless, these technologies alone are not responsible for such benefits. Using tablets or smartphones in a way that actually contributes to increased independence and participation is the main consideration (Lussier-Desrochers et al., 2017; McNaughton and Light, 2013; More and Travers, 2012). For example, previous research has frequently highlighted the need for adapting support for each individual using the technologies (McNaughton and Light, 2013; More and Travers, 2012; Näslund and Gardelli, 2013; Ramsten et al., 2018; Sorbring et al., 2017). The Australian study that examined the use of mobile technologies by disabled young adults concluded that those who get the most out of using mainstream technologies (e.g. for communicating and socializing with family and friends) are those who have significant ongoing support (training and technical adaption included therein) (Darcy et al., 2016).

Thus, people who support the young person’s use of ICT play a prominent role in constraining and/or enabling the benefits that might come with using these technologies (Barlott et al., 2019). As Clifford Simplican et al. (2018) highlight, the support staff’s attitude towards, idea of and knowledge about how to use new technologies is, therefore, important for people with intellectual disability. As emphasized in the literature, there is, nonetheless, a lack of research thoroughly examining how the social context shapes the way people with intellectual disability use ICT (Barlott et al., 2019; Clifford Simplican et al., 2018; Ramsten et al., 2018). Making progress in this area requires more than just educating the staff for a couple of hours and informing them of the possibilities that might open up with a new technology. Since the use of ICT takes place in a specific setting, it is the support staff’s ability to successfully adapt the new technology to the practices of the setting that matters.

Thus, this article deals with the way tablet devices become integrated into social care settings for youths with disabilities. The specific aim is to examine how staff members and the everyday practices of social care settings shape the way tablets are used. If and how do they create the conditions for enhancing the participation of young people with intellectual disability?

In the present context, participation is a person’s involvement in everyday life situations (WHO, 2007). People with disabilities may personally experience involvement when, for example, they feel listened to, are able to make their own choices or feel a sense of belonging and engagement (Arvidsson et al., 2008; Byhlin and Käcker, 2018). At the same time, to experience such involvement in daily situations, certain conditions must exist. According to Molin (2004), these conditions can be classified as internal (relating to the person’s ability and will to participate) and external (relating to opportunity and availability). These can be organized and planned in advance by support staff or they can arise in their interaction with the child/youth (Molin, 2004). With regard to this classification, our study deals with whether and how the staff create the external conditions for participation when using tablet devices in social care settings in Sweden.

Previous research has predominantly examined the conditions for young people with disabilities who use ICTs in the home and school and/or their experiences of said use (Barlott et al., 2019; Lidström and Hemmingsson, 2014; Molin et al., 2015; Sankardas and Rajanahally, 2017; Söderström and Ytterhus, 2010). Thorough examination of the way digital technologies are integrated into institutional settings other than the school has received less attention.

In accordance with the Swedish Disability Act concerning Support and Service for Persons with Certain Functional Impairments (LSS; SFS, 1993:387), people with disabilities such as intellectual disability and/or autism spectrum disorder (ASD) are entitled to special support. The overall objective of LSS is that, despite their disabilities, those receiving support should be able to participate in similar activities as their non-disabled peers.

The study presented in this article is based upon the staff’s experiences and how they shape the way iPads are used by youths with intellectual disabilities in LSS settings. Three types of LSS settings are considered: short-stay facilities, daily activity centres and housing with special services (SFS, 1993; Sjöberg, 2016). The first of these offers leisure activities, socializing with peers and/or relieving ordinary caregivers. Daily activity programmes tailored to individuals are provided in special centres or ordinary workplaces. Typical activities in daily activity centres include not only simple industrial tasks and different types of handicrafts but also occupational and physical therapy (Sjöberg, 2016). Housing with special services offers individually adapted support in residential arrangements, such as group housing with one’s own accommodations, that often include common areas and where there are staff nearby. To study how tablet devices are integrated into these LSS settings, we are guided by the concept of domestication.

## Technology domestication

Domestication deals with how technologies are integrated into, and used in, the everyday lives of users. This concept was originally developed in the 1990s (in the research undertaken in media and consumption studies) to help us understand how ICTs and ICT services are experienced in everyday life (Lie and Sørensen, 1996; Silverstone et al., 1992). Since then, the concept has been used and further developed in various studies (e.g. Berker et al., 2006), including examinations of the extent to which, in private homes, there has been domestication of widespread assistive technologies, such as beds, lifts and trapezes (Brodersen and Lindegaard, 2014), the more technically advanced telecare technology (Pols and Willems, 2011) and robots (Frennert, 2016).

The core of domestication is that the process of integrating technology into the everyday lives of users is dynamic and changeable. It is dynamic because the users shape the way technologies are used and, in the long term, future technological development (Silverstone et al., 1992). As the literature emphasizes, users do not simply adopt and accept technologies. Users are not passive receivers or ‘rational’ individuals acting exactly as technology producers intended (Lie and Sørensen, 1996). Sørensen (2006) describes domestication as a process that involves three interrelated dimensions: practical, cognitive and symbolic. The practical dimension centres on the concrete use of technology (how it is used and for what purposes), while the cognitive dimension revolves around learning (i.e. to be used effectively, technologies are objects that must be interpreted and made comprehensible). The symbolic dimension includes how people construct the meaning of the technology. It is a process of making the technology meaningful in their life. For instance, enhanced participation might (or might not) be what the individuals create using the technologies.

The relationship between users and technologies is not static. It changes over time. Not only does the initial, often experimental, use of a technology gradually evolve; early expectations and ideals also undergo modification. Domestication of new technologies is not necessarily a smooth process. For example, there may be various views on how to use a technology. Its purpose and what it brings to a certain setting may also be reviewed and reassessed over time (Sørensen, 2006).

Domestication takes place in a specific setting. As Pols and Willems (2011) point out: ‘New technologies are employed within particular practices, where users transform the technology to fit them into their own routines and goals’ (485). The study presented in this article describes in particular how tablet devices become domesticated in the LSS-related work settings of daily activity centres and short-stay facilities. Examples of common use practices in these settings include cooking, playing, training, handicrafts and social gatherings. As stated in the quotation, these practices have their own routines and goals; there are norms of how a practice such as cooking ought to be performed, and one needs certain knowledge and often various technologies and/or objects to carry out a practice (Reckwitz, 2002; Shove et al., 2012). On the one hand, the routines and goals of a certain practice shape the way technology is used, and on the other hand, domestication of technologies might change how a practice is carried out.

In line with the concept of domestication, the tablet devices are regarded as *integrated* when there is a recognizable and recurrent pattern of use that is fitted into the ordinary structuring of everyday practices in the social care settings. However, this article also gives examples of situations where individual youths recurrently use tablet devices in the settings.

The staff members’ intentions concerning the various ways of using the tablet devices are examined, and the meaning created by technology use, in terms of conditions enhancing the participation of youths with intellectual disability, is explored.

## Method

### Context of the study and participants

The study presented in this article is part of a 3-year (2016–2018) project involving the governmental organizations providing LSS support in six municipalities in western Sweden. The project’s main focui were: expanding the possibilities for using ICTs in LSS settings; involving members of staff in training; and providing tablet devices for the settings. The intention was to help increase the independence and enhance the participation of children and youths (people up to the age of 30, henceforth termed ‘youths’) in LSS settings. The whole project includes interviews with staff members as well as youths who were staying in the settings.

The empirical data presented in this article are based on interviews with the staff working in one of the participating municipalities that, in its LSS settings, had systematically implemented the use of iPads and associated programmes. This municipality was chosen since, thanks to a prior initiative (started in 2014), it already had experience of using digital technologies in said settings. Comprising both rural and urban areas, the municipality had approximately 13,000 inhabitants. In this municipality, the LSS settings had 80 individuals enrolled in LSS activities. Approximately half of these people were under 30 years of age.

We have interviewed staff members in three types of LSS settings: short-stay facilities, housing with special services and daily activity centres (SFS, 1993; Sjöberg, 2016). For inclusion in this study, the staff members had to work in any of the aforementioned settings.

The interviewed staff had been trained as nursing assistants, teachers or social workers. They worked full or part time. Eight of them worked in daily activity centres, four in short-stay facilities and four in housing with special services. One staff member (P3/9) was interviewed on two occasions because the first occasion was interrupted due to an incident at the setting. The letter P and a number from 1 to 17 are used to identify quoted participants.

**Table:** 

Settings	Participants
Daily activity centre	P1, P2, P6, P7, P8, P11, P14, P17
Short-stay facility	P3/9, P4, P5, P10
Housing with special services	P12, P13, P15, P16

To learn how to use a tablet device and various applications, most of the participating staff had attended at least one workshop. Many of them had also attended a workshop on using the Book Creator app. Different kinds of games (most of these being originally aimed at young children), crosswords, music apps and YouTube videos were the resources that were used. Tablet devices (iPads in particular) were the digital technology used at the institutional settings. Each setting had a couple of iPads available for use. Some of the youths also brought their own tablet devices.

### Procedure

The study used semi-structured interviews according to Kvale (2009). Information about the study and invitations to participate were orally communicated to staff members by representatives of the project group. From September to December 2016, 17 semi-structured interviews were conducted with 16 staff members.

During the interviews, the participants were asked to give detailed descriptions of up to three common situations in which one or more youths used the iPad in the setting in question. Most of the discussions in the interviews revolved around these situations. Follow-up questions were continuously asked regarding why they used the technology in the described ways and the interaction between the people involved in the situation (including how they assisted youths with the technology). One limitation of this procedure was that the participants mostly described situations where they were present and hence overlooked situations where youths used digital devices more independently in the setting.

The youths described in the situations were aged from 5 to 30 years and had, according to the staff, different types of intellectual disabilities, with or without ASD or physical impairments. The majority had moderate or severe impairments. The interviews lasted from 50 to 70 min. With consent, all interviews were audio-recorded and transcribed *verbatim* by the authors or an authorized transcriber.

### Data analysis

The interview data were analysed using thematic analysis. This is a ‘method for identifying, analysing and reporting patterns (themes) within data’ (Braun and Clarke, 2006: 79). As Braun and Clarke highlight, thematic analysis is often both inductive (data-driven) and deductive (guided by theory). Thus, in line with this method, both authors of this article read and reread, the transcribed interview manuscripts and subjected them to a detailed coding of words and phrases. The analysis was guided by the concept of domestication and divided into two phases. In the first phase, the notes taken from the interview transcripts centred on the various ways of using the iPads in the three settings and the specific intentions, as highlighted by the staff, behind such use (in particular the practical dimension of domestication). Also, as the study dealt with how iPad use had been integrated into the settings, there was an explicit search for incorporation, that is, patterns that were recurrent and adapted to everyday routines. To qualify as a pattern in the empirical data, technology use in the setting in question had to be recurrent and ongoing for at least a year.

Such systematic use of the technology was partly lacking in the interview transcripts of staff members working in houses with special services. These four interviews were still included in the analysis, and a few references are made to them in the text (for instance, when it comes to the non-systematic use of the iPad or general opinions of technology use their views did not deviate from the rest of the staff). However, they were not in focus in the recurrent coding procedure of the final main themes.

The empirical data from the other two settings (short-stay facilities and daily activity centres), offered patterns that satisfied the criteria and thus provided the basis for continued analysis. The coding that was employed identified two major themes: ‘Creating a new practice – the iPad sessions’; and ‘Using iPads in existing practices’. These are further presented in the results.

The second phase of the analysis revolved around exploring what ways the content of these themes (approaches) created conditions for the enhanced participation of youths with disabilities (symbolic dimension). This is presented in the discussion.

### Ethical considerations

The data collection for the study presented in this article was carried out in accordance with the WMA Declaration of Helsinki (2013). In connection with the interviews, all participants received oral and written information about the study. This included information on the rules of confidentiality and on the voluntary nature of participation. Participants gave written informed assent.

The study did not involve collecting sensitive personal data. When the staff members describe how various youths used the iPad, they did not reveal specific data about the youths, except an approximate age and impairments. Thus, this study does not analyse the individual abilities and/or characteristics of either the youths or the staff members involved. According to the Swedish Ethical Review Act (SFS 2003:460; 2018:147; 2018:1092), and after consulting a scientific secretary on the former Regional Ethical Board (today Swedish Ethical Review Authority) in April 2016, no approval was needed for the study presented in this article.

## Results

The findings reveal two overarching approaches for integrating tablet-device technology into the institutional settings. In the first approach, iPad use became a practice alongside others such as handicrafts, excursions and cooking. In the second approach, the technology was integrated into and used in already existing practices of the setting.

### Creating a new practice: ‘The iPad sessions’

In the settings, most of the daily activities were pre-organized and individually scheduled by staff members (sometimes in consultation with the youths). The iPad use was adapted to the existing individual schedules of the setting. The quotation below about a youth who spent her day at a daily activity centre is one illustration of how schedules were communicated to the youths:

Her schedule is on the wall. One picture shows her holding the iPad. She knows that this is when it’s time to work with the iPad. She works with it once a week. (P11)

Thus, the staff had invented a new practice, namely ‘using the iPad’. This was usually scheduled for 15 min to 1 h, one to three times a week. Socially, each session entailed one youth and one staff member sitting together, with no other people present, in a separate room. This arrangement was well-established and had not been subject to any changes. However, how staff and youths used iPads in the sessions changed slightly over time. In the beginning, the focus was on becoming familiar with the device and, by practicing the required sensorimotor skills and cognitive abilities, creating the right conditions for enabling the use of the digital technologies. Later, the sessions were primarily aimed at working with various apps to develop the youths’ language and mathematics skills. The staff also mentioned the unstructured use of iPads. Such use was scheduled but occurred during the time of the day when the youths had free activities. In these cases, iPad use was often initiated by the youths to entertain themselves. The following subsections give an insight into the various purposes and views of using iPad technology.

#### Promoting iPad familiarization by capturing interest

Staff members stated that capturing interest in iPad use is the first step. Initially, this was largely done using apps aimed at young children, for example, picture books where touch could be used to make animals produce noises, stars sparkle, leaves fall from trees and so on. The youths became aware that something happened when they touched a star, a leaf or an animal on the iPad. They experienced the ability to get the technology to do things. In the various settings, a youth’s ability to handle the iPad depended on the type of disability and the extent to which he or she was already familiar with the tool. Not every youth was able, or dared, to touch the screen in the beginning. The process of becoming familiar with the first steps of handling an iPad (e.g. touching stars that could sparkle) involved both overcoming the worry of handling something unknown and learning how to coordinate hands in the right way:

In the beginning, I was the one who had control. As he found it a little scary, he held my hand and I touched the iPad screen. However, he then started to move my hand. Later on, he used his own. Now, he controls it himself. Sometimes he wants us to participate. Sometimes it’s enough that we are by his side. (P2)

As the quotation illustrates, the youth gradually became familiar with the new tool. To begin with, the staff member demonstrated what to do. The youth followed the procedure closely. Subsequently, he could manage it by himself.

#### Using ‘working apps’ to improve skills

The apps used for improving language and mathematics skills varied with the ages, abilities and interests of the youths. Tablet apps featuring crosswords, jigsaws, pictures, colour learning, spelling programmes and children’s problem-solving games were used. It was the youth who, assisted by staff, worked with the app:

She usually works with the moderate crosswords, but can manage the difficult ones. Those take a little longer. We usually sit at the table in a secluded room every Tuesday afternoon. It is on her schedule. (P11)

As expressed by some of the staff, the main values of using these apps were improvement of not only language or mathematics skills but also of the ability to handle and manage new tasks:

He has expanded his knowledge of apps…Earlier, he didn’t want to try out new apps, but he does now. I believe he is trying out much more now. (P6)

Distinguishing them from other types of apps that were not considered particularly appropriate (e.g. an app involving repeatedly crashing a helicopter, a favourite with one of the youths), apps for improving skills were sometimes labelled ‘working apps’. As recounted by some staff members (P7), conflicts about how to use the technology sometimes arose during sessions. One of the solutions mentioned for this was to save a little time for entertainment at the end of working app sessions.

#### Being entertained

Using iPads for playing games and watching YouTube movies was, according to the staff, popular with the youths. This was primarily initiated by the youths themselves and occurred during the time scheduled for free play. For instance, one staff member mentioned a particular youth at a short-stay facility. Every time he saw the iPad, he wanted to use it for entertainment. As this prevented him from participating in other activities, the staff member restricted iPad access by putting it out of sight:

He does not want to build with Lego, chill with water or maybe watch movies. However, as it’s so immediate, he does enjoy the iPad…It’s like ‘him and the iPad’. (P10)

For this youth, using the technology brought a type of enjoyment that many other activities in the setting obviously did not. According to the staff member, iPad use relaxed the youth and got him to focus on one thing. However, because the various ways in which apps could be used often made it difficult to concentrate on one activity at a time, the technology had to first be adapted to the youth:

He used to spend 10 s on one app and then move to the next. (P4)

To prevent such movement, the staff usually locked in a particular app. In other words, the staff enabled the use of a particular app by limiting access to the variety of ways of using the iPad.

#### Different views on how to use the technology

In the social care settings, the ‘iPad sessions’ detailed above were the most common way of using iPads. Even though most of the staff members spoke favourably of the investment in and implementation of the technology, it was also clear that some of them expected more. This related to, on the one hand, the content of iPad sessions. As mentioned, the content changed slightly over time. However, some staff members explicitly stated that they had expected greater change. There were stories of youths still ‘stuck on testing’ various apps related to the basic step of handling the technology, for example, what happens when pressing on a leaf or a star (P2, P5). When the staff spoke of the expectations and possibilities of the technology, they tended, on the other hand, to talk about it as a tool for enhancing the youths’ participation in the existing practices of the setting. In this case, many staff members stated that increasing the youths’ opportunities for communication was regarded as a cornerstone in enabling participation in daily practices:

Almost none of them use verbal communication. They use easy kinds of sign language or single, spoken words. Thus, communication is important, and an iPad could really be a tool to facilitate communication. (P11)

How iPads were used to facilitate communication in relation to the existing practices of the settings will be presented next.

### Using iPads in existing practices

The staff described various ways in which they, together with the youths, had tried using iPads in existing practices. For instance, one staff member (P4) told us about using an application designed to facilitate cooking. Another (P13) recounted that, for youths living in housing with special services, work had just started on putting together pictures showing how to clean rooms. A third member (P8) detailed putting together, at the request of a youth doing carpentry at a daily activity centre, a book illustrating different kinds of tools to help with the youth’s woodworking. However, at the time of the interviews, none of these were well-integrated into the settings.

The following section gives an insight into integrated and hence habitual ways of using iPads in the existing practices of daily activity centres and short-stay facilities. Particular use was made of three iPad apps: the camera, a text document and/or the Book Creator app. Improving the youths’ writing and picture-taking skills was not the idea behind using these programmes. Instead, it was to facilitate interpersonal communication: informing the youths and preparing them to, as well as documenting and communicating about, everyday activities at the settings. This is demonstrated in the first two sections. The last section demonstrates situations when the use was not planned or organized by the staff members. It describes how the youths themselves repeatedly took the initiative in using iPads and applications (e.g. Google and pictures taken by the camera) to share events with others.

#### Facilitating information and preparation

As illustrated in the quotation below, the staff prepared participation in new activities outside the familiar social care setting (e.g. visits to libraries, forests, or nearby stores) by taking pictures in advance:

Especially for new activities, so that they will know what’s going to happen. For example, when we’re going to take a walk in the forest. Then, we take pictures showing where we can park the car, how we can find main paths, where we can walk, where we can turn off main paths, etc. (P4)

Using the Book Creator app, the staff put pictures in order and showed them to youths before new activities took place. According to the staff, the intention was to inform youths and prepare them for what was going to happen (thereby decreasing any potential frustration for the youths about which way to go, what to bring and how to perform the task). The pictures taken in advance demonstrated the norms and rules of a particular activity. As demonstrated below, providing advance information about ‘responsibilities’ was said to facilitate ‘correct’ participation in an activity:

He would then know how he was getting there and what was expected of him when he was there. For example, having to pay when renting a movie. (P5)

Additionally, staff members regarded going through the pictures with the youths as an opportunity for the youths to enhance their engagement (the possibility of not participating included therein) in the upcoming activity:

We wanted to prepare him so that we weren’t just imposing: ‘We’re going to do that; you’ll be coming along.’ We wanted him to feel he was part of the planning […] He thus knew what the plans were and could choose. He could also simply choose to do something else. (P4)

In the settings, youths were normally able to choose between different activities. Using photos enabled the youths to make informed choices, that is, they had a better picture of what the staff had in mind with the new activity.

#### Documenting events

The camera app was also used to document the events in a day. The quotation below relates to a youth at a daily activity centre. It illustrates greater involvement in picture-taking:

He uses his own [iPad] during the day in different activities. He always has it in his backpack. He uses it to take photos during activities or when he simply wants to take a picture. For example, he may see a bird that he wants to take a picture of. He indicates this to me, I hold the iPad and he pushes the button. (P7)

At the end of each ‘document day’, there was a scheduled session where youths and the staff members went through the pictures together and, using Book Creator, made a diary. The main goals of these sessions were to summarize the day and to support remembering what had happened. Communication between staff members and youths was facilitated by the photos. However, as recounted by some staff members, not all youths were interested in going through the day in this manner. The interest was more in subsequent communication with close relatives or friends who did not already know about the day:

When he returns home, he shows what he has done at work that day. He likes to share what he has done. (P7)

However, at the LSS setting, such communication with relatives, friends and other youths was not systematically organized. There was no certainty that, outside the setting, youths would actually have the possibility of using the picture diary to narrate their experiences:

I really don’t know if, at home, he takes any initiative in showing the I-book. I understand there is access to it. However, I really don’t know if he shows it or not. (P11)

#### Sharing events with others

Situations that were not planned and organized by the staff primarily revolved around youths using iPads as a tool for sharing meaningful events with others. A youth who participated in a daily activity group provided one example. A staff member recalled one incident when they were talking about what they were going to do during an upcoming summer vacation. The youth could not verbally tell the others about an imminent holiday trip with his family. This frustrated him. However, after a while, he scurried away and retrieved an iPad from the office. Despite incorrect spelling, he managed to google the travel agency’s site and was thereby able to show all the details:

All participants in his group were there when he told us about his trip. He could communicate with them as well as with us, the staff. This had a ripple effect. It has made him pick up the iPad often now…Although he does not want to use pictures (because it’s ‘childish’), but the iPad is ‘awesome’. (P8)

Another example is related to a youth at a daily activity centre. He liked to discuss the daily news. A staff member recounted that, before the introduction of iPads, such conversations were time-consuming and difficult because the youth had to use an ordinary newspaper. This had changed:

Now, when he wants to talk about the news, he gets the iPad, opens Aftonbladet [a Swedish newspaper] and scrolls to a news item. (P1)

Everyone, even those who are younger, becomes engaged when he tells you about the news. (P17)

In both these examples, the way the youths used the iPad had become a habit. As intimated, iPad use was continuous because it was convenient (the youths could more easily make themselves understood) and more socially acceptable than pictures. Besides, and as indicated in the last quotation, the interest shown by the youths who were listening was also important. A final example, which involves a female youth at a daily activity centre, especially highlights the impact of other people’s engagement. The youth had her own iPad and was often helped, by her sister and parents, to document various personal events. However, she seldom brought the iPad to the centre where she worked. One of the interviewed staff members recalled one particular time when the youth had brought the iPad and, with the support of the pictures that had been taken, related a car accident she and her family had been involved in. The staff member described the response of listening youths as overwhelming:

‘Oh God, how are you? Oh my God is everybody well? How do you feel?’ There was great contact. The others really showed her that they felt sorry for her. (P6)

Thus, the improved possibilities iPads offer for sharing events with others also make it easier for listeners to express liking and curiosity. The interviewed staff member stated that, after this incident, the youth started to bring the iPad more often to share other events and happenings.

## Discussion

The study’s findings reveal two overarching approaches for integrating iPad use into social care settings: creating a new practice and using iPads in existing practices. They both contain recognizable and recurrent ways of using the iPads that are fitted into the ordinary structuring of everyday practices in the LSS settings. Thus, they also demonstrate two different ways of domesticating technology in the setting, which might have quite different implications for the youths using the technology. In the following, there is a discussion of the practical and cognitive and, in particular, the symbolic dimensions involved in each approach (c.p. Sørensen, 2006). This latter dimension is addressed in a reflection on how the various approaches enhance the conditions for youth participation.

The first approach detailed in this article was the most common. It involved staff members creating a new practice: ‘using the iPad’. This was often scheduled. Aims included promoting iPad familiarity, language and mathematics skills, and entertainment. Viewed purely in terms of participation, this approach to iPad incorporation might be interpreted as a failure. It did not directly create opportunities for enhancing youth participation in the everyday practices of the setting. Rather, iPad use *replaced activities* such as reading books, drawing on paper and playing with ordinary toys. Furthermore, through the promotion of working apps, the staff often determined *how* the iPads ought to be used during the sessions and the scheduling by the staff determined *when* iPad use should occur. A common description of assistive technology is that it is close at hand and can be used as a facilitator for participation in several different situations (Cook and Polgar, 2015). However, using an iPad on Tuesdays and Thursdays between 14:00 h and 15:00 h is a far cry from this goal. Similarly, having only two or three iPads per setting is insufficient.

Still, there are also other interpretations of the first approach. In fact, scheduling use of the technology could be a way to ensure it is used regularly and not forgotten (c.f. Brodersen and Lindegaard, 2014; Harris, 2010). Through the iPad sessions, the staff member also created the conditions for youths to increase their ability to handle the iPad and its various applications. This simultaneously provided opportunities for working on the sensorimotor and cognitive obstacles people with intellectual disability often face with such technologies (Chadwick et al., 2013; Lussier-Desrochers et al., 2017). In addition, when youths could choose, playing games and watching YouTube were their preferred activities on iPads. This gave them access to digital activities commonly used by their non-disabled peers. These conditions could be seen as a way of mitigating digital exclusion in society (Macdonald and Clayton 2013) and a step towards increased participation and independence in the everyday practices of social care settings.

Domestication of technologies is seldom a linear process. Conflicts regarding technology use and purpose are common (Berker et al., 2006; Lie and Sørensen, 1996; Silverstone et al., 1992). For example, as here recorded, youths and staff members did not always agree on how the technology should be used in iPad sessions (games or educational purposes). Staff members’ views on iPad use in each setting also sometimes diverged. Some of the staff members highlighted stagnation in the iPad session. Others spoke of the possibilities for using the tool to enhance communication and social participation in activities carried out in the setting.

In the second approach, with the overall purpose of facilitating communication, iPad use was adapted to the existing practices of the setting. This study’s findings detail how, by enabling the use of iPads and various applications, the staff created opportunities for youths to make informed choices, thereby enhancing their engagement. The importance of striving for this kind of agency are illustrated in previous literature examining disabled people’s experiences of participation (Arvidsson et al., 2008; Byhlin and Käcker, 2018; Ramsten et al., 2017). However, a further aim of iPad use was also to illustrate expectations of how an upcoming activity should be carried out (thereby creating conditions for youths to better conform to the norms and rules of a particular practice). This raises issues of *who* the main users really are and who actually benefits from using the technology in the intended way (Brodersen and Lindegaard, 2014). Staff members, whose work might be easier if an activity goes smoothly, or the youths learning to emulate the normal behaviour of the non-disabled majority?

This study’s findings also detail how staff members and youths together used iPads to document their days. In separate, scheduled sessions, they summarized events that had taken place during the day or, in other words, made personal diaries. However, this was often where the details of this activity ended. There were not any organized opportunities for the youths to use their diaries later on for sharing events with others in the social care settings or at home.

Cumming et al. (2014) describe how photographs taken with the iPad’s camera helped a non-verbal person to communicate and share events with others. It was not just that the pictures and photographs gave this person concrete cues for conversation. The person additionally became more interesting to listeners who, in turn, spent more time communicating with the person. Some of the youths at the settings used the technology for purposes similar to the aforementioned example. In fact, the most enthusiastic descriptions of how the technology was used and could be used in the future concerned instances of youths creating ways of using the iPad to communicate with others (or taking the initiative for doing this). That the youth made a habit of using the iPad for this purpose also indicates that it was an acceptable and preferable device for communicating with others. This is also found in a study done by Barlott et al. (2019) that examined how 10 people with intellectual disability used ICT. They demonstrated that participants with verbal communication difficulties preferred the smartphone or iPad to augment their communication rather than using a dedicated communication device. Thus, they used the same technologies as their non-disabled peers and avoided the stigmatization that has been identified in relation to dedicated communication devices (McNaughton and Light, 2013; Söderström and Ytterhus, 2010).

Svanelöv et al. (2019), who explored participation in daily activity service among people with intellectual disability, highlight that social interaction is a prerequisite for participation. Thus, when the staff members in our study addressed the importance of using the iPad as a tool for facilitating communication, they highlight an important condition for participation. However, a focus on using the iPad for facilitating social interaction is not enough. In addition, the staff need to consider whether the interaction achieved with the iPad actually creates a feeling of involvement from the perspective of the youths. Continuous input from the youths on these matters is then a necessity. Moreover, we argue for the importance of paying attention to the collective benefits that might come with using an iPad or a smartphone. For instance, when single youths used the iPad to show their own photographs and pictures taken from websites, they also increased the opportunities for listening youths to get involved.

## Conclusions

The specific aim of this article has been to examine how staff members and the everyday practices of social care settings shape the way tablets are used. We have demonstrated two approaches to using the tablets. One conclusion is that the way the technology is fitted into the ordinary structuring of everyday practices in these settings matters: when iPad use was scheduled and became a practice alongside others (iPad session), it *replaced existing activities* (reading books, doing crosswords, etc.). When it was used in already existing practices, it became *a tool for facilitating communication.*

If and how did these approaches create conditions for enhanced participation of youths with intellectual disability? In line with the insights of previous research (Molin et al., 2015), another conclusion of this study is that staff members largely planned and created conditions for youths to participate *like* non-disabled youths. Examples include using the iPad for educational purposes, playing games (iPad sessions) and preparing for participation in upcoming activities (in relation to existing practices). However, by integrating iPads into the settings, the staff also became aware of the technology’s potential for increasing the youths’ opportunities for participating *with* each other. The staff members described with particularly great enthusiasm situations where youths themselves spontaneously took the initiative and used the iPads for sharing events with others.

An additional conclusion is that there is a need for flexible as well as long-term strategies and goals that inform the effort to make the most of the technology by focusing on enhancing youths’ participation in social care settings. As the domestication process is dynamic, the initial goals and plans for how to use a technology should not be so fixed that change is precluded. Rather, as Pols and Willems state, ‘changing goals are to be expected and encouraged rather than avoided’ (2011: 494). In this study, adapting goals to what the youths regard as meaningful would, for example, include strategies for improving the conditions for these youths to use the technology for sharing events and communicating with each other.

### Implications for practice and future research

In our study, many of the situations described by the staff concerned youths diagnosed with a moderate or severe intellectual disability. In this case, the staff’s ideas and how they shape the way iPads are used in the setting, become very important, since the youths involved need more help using and organizing their use of the technology. To improve the services for people with intellectual disbilities, the staff need to reflect upon and test ways that enhanced participation may be achieved with the help of ICT. Simply implementing digital technologies in institutional settings does not necessarily lead to enhanced participation. It might only replace non-digital activities. However, using tablets as tools for facilitating interpersonal communication is a promising way forward. It is likely to enhance the social participation for the individual youths using the technology as well as for those youths who are listening.

As pointed out in the introduction, technological devices do not, in themselves, enhance the lives of people with disabilities. What matters is how they are used and integrated into the practices of particular settings and the benefits created by this process. To ensure that technological development benefits all those concerned and that resources are used as efficiently as possible, domestication processes must be examined and analysed more thoroughly.

The study presented in this article was limited to examining the experiences of staff members and how they shaped technology use. To enhance participation for young people with intellectual disabilities through the use of technological devices, input from the youths is needed. A second study has also been conducted within the project to examine technology use from the youths’ perspective. However, the staff members’ and youths’ separate views and actions in relation to technology use are still not enough. In fact, future research needs to address how staff members and youths can collaborate to enhance the youths’ participation.

## Note

1. Book Creator (https://bookcreator.com) is an application to create e-books on tablet devices.
